# The impact of germline BRCA pathogenic variants in locally advanced, triple negative breast cancer treated with platinum-based neoadjuvant chemotherapy

**DOI:** 10.1007/s10549-024-07247-4

**Published:** 2024-02-12

**Authors:** Raz Mutai, Iryna Kuchuk, Alexandra Goldshtein, Rinat Yerushalmi, Ofer Rotem, Adi Maisel Lotan, Tali Bdolah-Abram, Alberto Gabizon, Hadar Goldvaser

**Affiliations:** 1 Davidoff Cancer Center, Rabin Medical Center, Petah Tikva, Israel; 2 Faculty of Medicine Tel-Aviv University, Tel-Aviv, Israel; 3 The Oncology Institute, Meir Medical Center, Kfar Saba, Israel; 4 Department of Military Medicine and “Tzameret”, Faculty of Medicine, Hebrew University of Jerusalem, Jerusalem, Israel; 5 Medical Corps, Israel Defense Forces, Ramat Gan, Israel; 6 Plastic Surgery Department, Shaare Zedek Medical Center, Faculty of Medicine, Hebrew University, Jerusalem, Israel; 7 Faculty of Medicine, Hebrew University, Jerusalem, Israel; 8 The Helmsley Cancer Center, Shaare Zedek Medical Center, Faculty of Medicine, Hebrew University, 9103102 Jerusalem, Israel

**Keywords:** Breast cancer, Neoadjuvant chemotherapy, BRCA mutation, Triple negative

## Abstract

**Background:**

Whether germline BRCA (gBRCA) pathogenic variants (PV) affect prognosis of women with triple negative breast cancer (TNBC) and whether it has implications for treatment decisions in the neoadjuvant setting is unclear.

**Methods:**

This is a retrospective two-center cohort study comprising all women with early stage TNBC who have completed genetic testing and were treated with neoadjuvant dose-dense doxorubicin and cyclophosphamide followed by paclitaxel and carboplatin. All eligible patients treated between 10.2014 and 3.2020 were included. Data on clinico-pathological, pathological response, overall survival (OS) and disease-free survival (DFS) were evaluated. Differences in clinico-pathological features and outcomes were analyzed according to gBRCA status.

**Results:**

Sixty-four women were included in the final analysis, of which 31 had gBRCA PV (gBRCA carriers) and 33 were gBRCA wild-type. Clinico-pathological characteristics were similar between both groups. The odds for pathological complete response (pCR) were significantly higher in gBRCA carriers (74.2%) compared to BRCA wild-type women (48.5%), *p* = 0.035. At a median follow-up of 30 months, gBRCA carriers had significantly favorable OS (HR = 8.64, 95% CI 1.08–69.21, *p* = 0.042). The difference in DFS did not reach statistical significance (HR = 7.4, 95% CI 0.91–60.27, *p* = 0.062). The favorable OS for gBRCA carriers remained significant in multivariate analysis (*p* = 0.029) and was noted regardless of pathological response (*p* = 0.018).

**Conclusion:**

Compared to wild-type, gBRCA carriers with locally advanced TNBC treated with neoadjuvant chemotherapy containing carboplatin had a higher pCR rate and better outcomes. These results strengthen the contention that gBRCA status should be considered when tailoring treatment decisions in women with locally advanced TNBC.

## Introduction

Triple negative breast cancer (TNBC) accounts for 10–15% of breast cancers and is associated with worse outcome compared to other subtypes [1, 2]. Pathogenic germline mutations are identified in approximately 10–15% of all patients with breast cancer, of which pathogenic variants (PV) in germline BRCA 1/2 genes (gBRCA) are most common [3]. In contrast to the distribution of breast cancer subtypes in the average population, BRCA1 carriers are more likely to develop TNBC, reaching over 50% of cases [4].

Except the recently approved adjuvant olaparib for high risk gBRCA carriers [5], the systemic treatment for early stage disease is identical for gBRCA carriers and wild-type breast cancer patients. However, current data suggest that breast cancer in gBRCA patients might represent a distinct entity with a different course of disease. gBRCA carriers with estrogen receptor (ER) positive, human epidermal growth factor receptor 2 (HER2) negative disease tend to have higher oncotype recurrence score than wild-type patients, supporting worse prognosis and potentially greater benefit from adjuvant chemotherapy [6]. Further investigation of this cohort has revealed gBRCA carriers have a distinct gene expression profile compared to the control group [7]. In metastatic TNBC a significant difference in response to chemotherapy was identified, with higher response to platinum therapy in gBRCA carriers, while BRCA wild-type patients had higher rates of response to taxanes [8]. gBRCA carriers with early stage TNBC achieve higher rates of pathological complete response (pCR) compared to BRCA wild-type, regardless to the addition of platinum to the neoadjuvant regimen [9, 10]. Although pCR in associated with improved outcome in TNBC it is not clear whether gBRCA is an independent predictor for survival [11].

Whether gBRCA carriers with breast cancer represent a distinct clinical entity and whether additional adaptations in their treatment might improve long-term outcomes is unknown. The aim of this study was to investigate the impact of gBRCA PV on outcomes in patients with locally advanced TNBC who are treated with platinum-based neoadjuvant chemotherapy.

## Methods

### Study population

Data were collected retrospectively from the electronical medical records of the Meir and Rabin Medical Centers, both large community hospitals and referral centers in central Israel. All adult women with early stage TNBC who were treated with neoadjuvant chemotherapy comprising doxorubicin and cyclophosphamide (AC), and carboplatin and taxanes regimen between 10.2014 and 3.2020 were identified. Patients who received pembrolizumab in addition to chemotherapy were also included.

Data of demographics and clinical-pathological features were collected including age, BRCA status (carrier, wild-type or unknown), histological subtype, clinical tumor size and nodal status and pathological staging. Of note, different methods of genetic testing are done in Israel for breast cancer patients: some undergoing only genotyping of familiar recurring gBRCA PV in Israel, while for other next generation sequencing (NGS) utilizing commercially available tests is done. Data on dose reduction and delays were also collected. An event of treatment delay was defined as administration of therapy more than 7 days after the pre-planned date. Long-term outcomes including loco-regional recurrence, distal recurrence and death were extracted from medical records. Disease-free survival (DFS) and overall survival (OS) were calculated. Data lock was in 11/2021. DFS duration was defined as the time between surgery to any event (recurrence or death) or date of data lock. pCR was defined as no residual invasive disease in both breast and lymph nodes. The study was approved by the institutional ethics committees in accordance with the declaration of Helsinki in both medical centers.

### Statistical analysis

Differences in patients' characteristics and outcomes between gBRCA carriers to wild-type patients were the pre-specified primary outcome. We also analyzed differences in dose density between both patient groups. Patients without known genetic status were excluded from the primary analyses. Data were reported descriptively for both groups. For categorical variables, the Chi-square or the Fisher's exact test was used. The comparison of a quantitative variable between two independent groups was performed using the two-sample t-test. Assessing the effect of categorical variables on OS and DFS was carried out using the Kaplan–Meier survival analysis model with the log-rank test for comparing survival curves. The effect of quantitative variables on OS and DFS was tested using the Cox regression model. This model was also applied as the multivariate model, for simultaneously assessing the effect of several variables on OS including age, clinical nodal stage (node positive vs. node negative), clinical tumor stage (clinical tumor size ≤ 2 cm vs. > 2 cm) and gBRCA status. This model yields *p* values as well as adjusted Hazard Ratios (HR) with 95% Confidence intervals (CI) for each of the variables entered into the model. All tests applied were two-tailed, and a *p* value of 0.05 or less was considered statistically significant. Statistical analysis was carried out using the IBM SPSS Statistics program, version 28.

## Results

Overall, 97 patients were identified. After exclusion of 33 patients with unknown BRCA status, 64 patients were included in the final analysis, of which 31 were gBRCA carriers and 33 were BRCA wild-type. The median follow-up time was 30 months.

The demographic and pathological characteristics are presented in Table 1. Median age was 43 (range 27–81), 50 (78%) presented with clinical T1-2 tumor (tumor size ≤ 5 cm) and 46 (74%) had node positive disease. Tumor characteristics were similar between gBRCA carriers and wild-type patients. Twelve patients were treated with pembrolizumab in addition to chemotherapy, 6 patients in each group.

**Table 1:** Patients demographic and clinical characteristics

Variable	All (*n* = 64)	BRCA wild-type (*n* = 33)	gBRCA carriers (*n* = 31)	*p* value
Age				
Median (range)	43 (27–81)	42 (27–76)	43 (29–81)	
≤ 50	44 (70%)	22 (69%)	22 (71%)	0.79
> 50	20 (30%)	11 (33%)	9 (29%)	
cT				
1–2	50 (78%)	24 (73%)	26 (84%)	0.37
3–4	14 (22%)	9 (27%)	5 (16%)	
cN				
0	18 (26%)	10 (30%)	8 (26%)	0.78
1–3	46 (74%)	23 (70%)	23 (74%)	
ypT				
0	41 (64%)	17 (52%)	24 (77%)	0.18
ypT1a-b	14 (22%)	10 (30%)	4 (13%)	
ypT1c	5 (8%)	3 (9%)	2 (7%)	
ypT2 or larger	4 (6%)	3 (9%)	1 (3%)	
ypN				
ypN0	53 (83%)	25 (76%)	28 (90%)	0.19
ypN positive	11 (17%)	8 (24%)	3 (10%)	
pCR-ypT0N0				
Yes	39 (60.9%)	16 (48.5%)	23 (74.2%)	0.035
Histological subtype				
IDC	63 (98%)	32 (97%)	31 (100%)	1
Other	1 (2%)	1 (3%)	0 (0%)	

*IDC*, invasive ductal carcinoma, *pCR* pathological complete response, *cT* clinical tumor size: T1 ≤ 2 cm, 2 < T2 ≤ 5 cm, T3 > 5 cm, T4 extension to skin or chest wall, *cN* clinical lymph nodes involvement, *ypT* pathological tumor size in patients who received neoadjuvant chemotherapy: T1a-b ≤ 1 cm, 1 < T1c ≤ 2 cm, T2 > 2 cm, *ypN* pathological nodal stage in patients who received neoadjuvant chemotherapy

Dose reduction rates were comparable between gBRCA carriers and wild-type for all types of chemotherapy including: AC (*p* for the difference = 0.19), paclitaxel (*p* = 0.51) and carboplatin (*p* = 0.39). There was a difference in paclitaxel treatment intensity with significantly more delays in paclitaxel therapy in gBRCA carriers (*n* = 22, 71%) compared to wild-type patients (*n* = 14, 42%), *p* = 0.02.

**Fig. 1:**
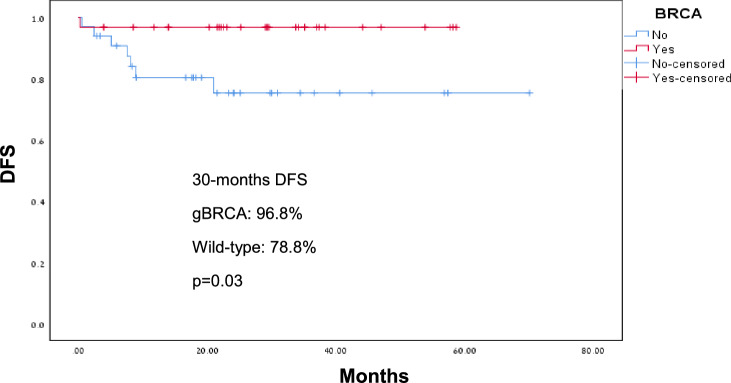
Disease-free survival for gBRCA carriers and wild-type BRCA

**Fig. 2:**
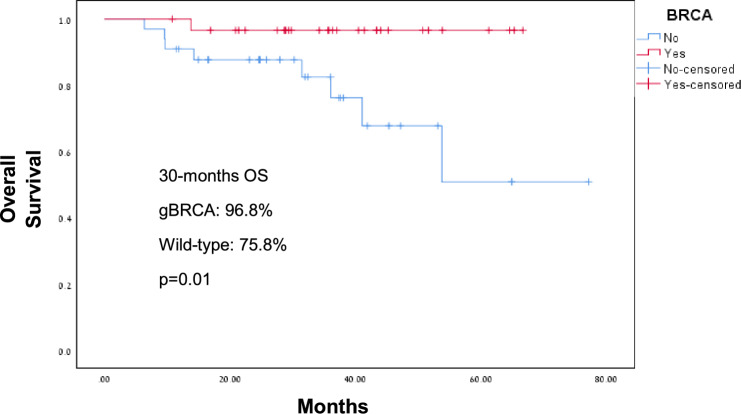
Overall survival for gBRCA carriers and wild-type BRCA

**Fig. 3:**
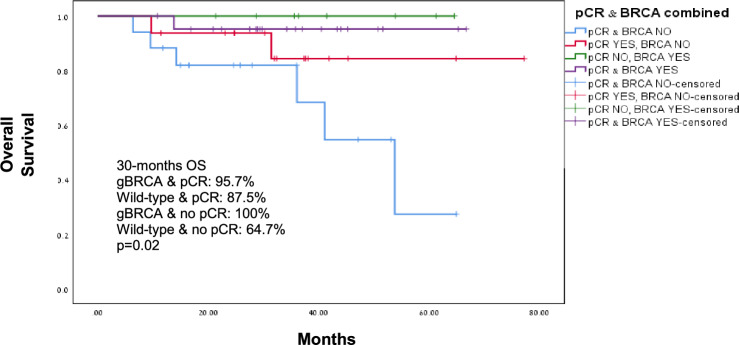
Overall survival by BRCA and pCR status

gBRCA PV were associated with significantly higher odds of achieving pCR (23 patients, 74.2%) compared to wild-type disease (16 patients, 48.5%), *p* = 0.035. The estimated DFS by Kaplan- Meier during the follow up was 96.8% and 78.8% for gBRCA carriers and wild-type patients, respectively (*p* = 0.02), Fig. 1. The HR for DFS approached statistical significance (HR = 7.39, 95% CI 0.91–60.27, *p* = 0.062). The estimated OS during the follow up time was significantly better for gBRCA carriers (96.8%) compared to wild-type patients (75.8%) with HR = 8.6, 95% CI 1.08–69, *p* = 0.014, Fig. 2. Subgroup analysis for OS by pCR showed better OS for gBRCA carriers regardless to whether they achieved pCR or not, *p* = 0.018 (Fig. 3). Furthermore, in multivariate analysis for OS including age, tumor size, lymph nodes involvement and BRCA status, the association between OS and gBRCA remained significant (*p* = 0.029), Table 2.

**Table 2:** Multivariant analysis of association between OS and age, lymph node involvement, tumor size and gBRCA status

Variant	Adjusted HR (95% CI)	*p* value
Age at diagnosis	1.004 (0.95–1.062)	0.877
gBRCA wild-type vs. carriers	10.717 (1.28–89.81)	0.029
cT1		0.516
cT2	NS	0.943
cT3-4	NS	0.939
cN positive vs. negative	2.316 (0.42–12.79)	0.335

*cT* clinical tumor size: T1 ≤ 2 cm, 2 < T2 ≤ 5 cm, T3 > 5 cm, T4 extension to skin or chest wall, *cN* clinical lymph nodes involvement, *CI* confidence interval, *HR* hazard ratio

## Discussion

This study investigated the differences in long-term outcomes according to BRCA status in patients with locally advanced TNBC who were treated with platinum-based neoadjuvant chemotherapy. gBRCA carriers had significantly better rates of pCR and OS, while age and other prognostic tumor characteristics were comparable between both groups.

The observation of improved pCR rates for gBRCA carriers in our cohort is consistent with previous results from both prospective randomized studies including the CALGB 40603, BrighTNess, and the GeparOcto studies and retrospective reports [10, 12–14]. To the best of our knowledge, our study is the first to show improved DFS and OS in gBRCA carriers compared to wild-type BRCA disease. While the association between pCR and improved outcome in TNBC is well established [15, 16], prior studies did not report explicitly that gBRCA carriers had improved outcomes. Indeed, in our cohort patients who achieved pCR had improved survival regardless to BRCA status, however subgroups analysis by pathological response showed that compared to wild-type BRCA, gBRCA PV were associated with improved OS also in women who did not achieve pCR.

Women who are aware of their BRCA status prior breast cancer diagnosis usually undergone intensified surveillance and are diagnosed in earlier stage [17]. In addition, BRCA carriers who are aware to their status prior to breast cancer diagnosis is also associated with improved survival compared to women that were identified with gBRCA PV subsequent to breast cancer diagnosis [17]. Data on the timing on the gBRCA diagnosis were not available in our study, however age and tumor characteristics were comparable between both groups. Additionally, multivariate analysis that included tumor characteristics showed that the association between improved OS and gBRCA PV remained robust.

The role of BRCA1 and BRCA2 proteins in the repair of DNA damage pathway raised the hypothesis that gBRCA carries have an increased sensitivity for chemotherapy, including both cancer as well as rapidly dividing cells such as bone marrow cells [18]. There are inconsistent data on the association between gBRCA PV and increased risk chemotherapy induced myelotoxicity [19–22]. In our cohort a significant decrease in paclitaxel intensity among gBRCA carriers was identified. This finding may further support the increase risk of gBRCA carriers for chemotherapy associated myelotoxicity, especially when given with DNA crosslinking agents, such as carboplatin. Interestingly, the inferior dose intensity did not have negative effect on outcome, despite the well-known prognostic role of dose intensity in early breast cancer [23]. Whether this discordance is related to a direct improved prognosis of gBRCA carriers in unknown.

Our findings suggest that breast cancer in gBRCA carriers represents a distinct clinical entity. This is supported by prior studies from both early and metastatic settings which reported that breast cancer patients with gBRCA PV have different response to therapy compared to BRCA wild-type patients [8, 9]. Currently, the only implication of BRCA status on decision-making in early stage disease is limited to escalating therapy in the adjuvant setting in selected high risk gBRCA carriers [5]. BRCA status is a potential target to better tailor therapy in early breast cancer.

Current standard of care for locally advanced TNBC comprises neoadjuvant combination of multi-agent chemotherapy and pembrolizumab [25, 26]. While this therapy improves breast cancer outcomes, it is associated with high rates of toxicity including long-term toxicity [24, 26]. As a unique patient population with an improved response to therapy, gBRCA carriers are good candidates for further research aiming to identify patients who might achieve excellent outcome with deescalated therapy. A single arm study has shown neoadjuvant therapy with single agent PARP inhibitor in gBRCA carriers achieved high rates of pCR, suggesting some patients with gBRCA PV might have excellent prognosis without chemotherapy, even in TNBC [27]. Ongoing clinical trials which investigate the efficacy of PARP inhibitors with or without immunotherapy in early stage gBRCA carriers will shed light on the role of chemotherapy free regimen in this population (NCT05498155 and NCT04584255).

Taxane-based regimen without anthracyclines has high efficacy and is associated with reduced life-threatening toxicity such as cardiotoxicity and secondary malignancy [28], but for TNBC combination chemotherapy with anthracyclines has remained standard of care [25, 29]. A recent study has shown that the combination of neoadjuvant pembrolizumab and six cycles of carboplatin and docetaxel for TNBC achieves high rate of pCR [30], similar to this of the KEYNOTE 522 study [24], suggesting that in the era of immunotherapy, a regimen without anthracyclines could potentially be appropriate also in TNBC. Considering our findings of higher response and favorable outcomes of gBRCA carriers with TNBC, prospective studies that will investigate the anthracyclines free regimen in gBRCA carriers is important.

In this cohort BRCA wild-type patients had notable high recurrence rate and only 75.8% survival rate after median follow-up of 30 months, despite treatment with 4 different chemotherapy agents. Escalating therapy in the BRCA wild-type patient population might improve their outcome. Adjuvant capecitabine in TNBC patients who did not achieve pCR to NAC chemotherapy has shown to improve survival, but relapse rate remains relatively high compared to other breast cancer subtypes [31]. On-going studies comparing adjuvant sacituzumab govitecan to standard of care (NCT05633654, NCT04595565) in this high-risk population may further improve outcomes. Additional investigation on escalated therapy in BRCA wild-type, locally advanced TNBC patients is important to further improve their outcome.

This study has several limitations. First, this is a retrospective study vulnerable to unknown bias or incomplete information due to partial documentation. However, we used hard endpoints such as OS, unlikely to be affected by documentation issues. Second, the study cohort is relatively small and caution should be taken when interpreting our findings. Of note, considering the fact that gBRCA PV are identified only in 5–10% of all breast cancer patients and TNBC is the least common subtype, 31 gBRCA carriers that were treated with a modern chemotherapy regimen is a meaningful sample size. Third, a selection bias is possible as patients who are candidates for neoadjuvant therapy comprising 4 different types of chemotherapy are expected to be fit and less likely to include older patients or patients with multiple comorbidities. Fourth, the method for genetic testing was not document, and some women performed only genotyping for familiar recurring gBRCA rather than NGS testing, potentially underdiagnosing gBRCA carriers in the wild-type cohort. Of note, in a previous study from Israel, most gBRCA PV that were identified in a multi-gene NGS panel were also PV that could be identified by genotyping only for familiar gBCRA PV [32]. As such, the vast majority of our wild-type BRCA cohort is expected to be truly negative. Fifth, data on adjuvant olaparib or capecitabine as well as on the duration of pembrolizumab therapy in patients who receive this therapy, were not available. This information could be valuable when interpreting our results, as these therapies are associated with significantly improvement in outcome [5, 31]. Adjuvant olaparib was FDA approved approximately 2 years after the last patient in our cohort was included, therefore there was probably no or minimal exposure to adjuvant olaparib in our cohort. Finally, the duration of follow-up is relatively short and longer follow-up might change outcomes results. However, as most recurrences of TNBC occur in the first 3 years after diagnosis, similar trends for improved DFS and OS are expected to be seen after longer follow-up. Importantly, only patients with known genetic status were included in our analysis and the chemotherapy that was consistent with a modern regimen adds strength to our study.

In conclusion, among patients with TNBC who received platinum-based neoadjuvant chemotherapy, gBRCA PV were associated with higher odds for pCR as well favorable DFS and OS. The improved OS was noted also in BRCA carriers who did not achieve pCR. Whether the initial therapy for TNBC in this population should be different from the standard treatment of the general population is unknown. Reporting of BRCA status in clinical trials on early stage breast cancer should be mandatory to further elucidate differences between gBRCA and wild-type disease.

## Data Availability

The datasets and analysis are available from the corresponding author on reasonable request.
